# Loss of TIMP-1 immune expression and tumor recurrence in localized prostate cancer

**DOI:** 10.1590/S1677-5538.IBJU.2014.0451

**Published:** 2015

**Authors:** Sabrina Thalita dos Reis, Nayara Izabel Viana, Alexandre Iscaife, José Pontes, Nelson Dip, Alberto Azoubel Antunes, Vanessa Ribeiro Guimarães, Isaque Santana, William Carlos Nahas, Miguel Srougi, Katia Ramos Moreira Leite

**Affiliations:** 1 Laboratório de Investigação Médica (LIM55), Departamento de Urologia da Universidade de São Paulo Faculdade de Medicina de São Paulo, Brasil; 2 Genoa Biotechnology SA, São Paulo, Brasil; 3 Instituto do Câncer do Estado de São Paulo, São Paulo, Brasil

**Keywords:** Prostatic Neoplasms, Matrix Metalloproteinases, Prognosis, Diagnosis, Gene Expression

## Abstract

**Introduction and objective:**

Overexpression of MMPs has been related to biochemical recurrence after radical prostatectomy. TIMP1 and TIMP2 are controllers of MMPs and the aim of this study is to evaluate the expression levels of MMPs and their regulators using immunohistochemistry in tissue microarray of localized prostate cancer (PC).

**Materials and Methods:**

Immune-expression of MMP-9, MMP-2, TIMP1, TIMP-2, MMP-14 and IL8, were analyzed by immunohistochemistry in radical prostatectomy specimens of 40 patients with localized PC who underwent surgery between September 1997 and February 2000. Protein expression was considered as categorical variables, negative or positive. The results of the immune-expression were correlated to Gleason score (GS), pathological stage (TNM), pre-operatory PSA serum levels and biochemical recurrence in a mean follow up period of 92.5 months.

**Results:**

The loss of TIMP1 immune-expression was related to biochemical recurrence. When TIMP1 was negative, 56.3% patients recurred versus 22.2% of those whose TIMP1 was positive (p=0.042). MMP-9, MMP-2, IL8 and MMP-14 were positive in the majority of PC. TIMP-2 was negative in all cases.

**Conclusion:**

Negative immune-expression of TIMP1 is correlated with biochemical recurrence in patients with PC possibly by failing to control MMP-9, an important MMP related to cancer progression.

## INTRODUCTION

Degradation of basal membranes and extracellular matrix (ECM) is essential for tumor invasion and development of metastases, and matrix metalloproteinases (MMPs) are potent proteolytic enzymes that are known to play a key role in these processes. Within the MMP family, Matrix Metallproteinase 2 (MMP-2) (gelatinase A, 72 kDa) and Matrix Metalloproteinase 9 (MMP-9) (gelatinase B, 92 kDa) cleave type IV collagen and gelatin, which are the main structural components of the basal membrane (1). MMP-9 and MMP-2 expression has been implicated in the development and progression of many tumors, such as bladder (2), colorectal (3), lung cancer (4) and prostate cancer (5).

MMPs are transcriptionally regulated. MMP-2 is mainly regulated by its zymogen inhibitor, tissue inhibitor of metalloproteinase 2 (TIMP 2), and by its major activator, membrane type-1 MMP (MT1-MMP), also known as MMP-14. MT1-MMP specifically activates the pro-gelatinase, MMP-2, on the tumor cell surface in vitro through the formation of a complex with TIMP-2 (6). IL8 upregulates MMP-2 in tumor cells, which is thought to be responsible for its angiogenic activity (7). MMP-9 is mainly regulated by TIMP-1 and has been reported that reversion-inducing cysteine-rich protein with Kazal motifs (RECK) inhibits both MMP-2 and MMP-9 (8).

The balance between secreted MMPs and their specific regulators plays an important role in the maintenance of connective tissue homeostasis in normal and pathological tissues (9). In neoplastic diseases, including prostate, an imbalance between MMPs and their inhibitors, leading to an excess of degradative activity, is assumed to be related to the invasiveness capacity of tumor cells (10–12).

In a previous study, we have analyzed the gene expression of MMPs and their regulators in PC by qRT-PCR, and found that MMP-9 was upregulated probably as a consequence of the under-expression of its negative regulators. Moreover, the levels of MMP-9 were higher in patients with preoperative PSA>10ng/mL, and most importantly in those who have presented biochemical recurrence (8). We also noted that TIMP-2, MT1-MMP and IL8 were overexpressed and would be possibly responsible for the decrease in MMP-2 expression in PC tissue (5).

To validate our previous findings, we decided to search for protein expression of MMPs and its regulators by immunohistochemistry in a tissue microarray representative of radical prostatectomy specimens of men followed by a mean period of 92.5 months, trying to find new prognostic markers for the disease.

## PATIENTS AND METHODS

### Patients

The study was conducted using surgical specimens from 40 patients with clinically localized PC (pT2/3N0M0) who underwent radical prostatectomy in our institution between 1993 and 2007. These cases were randomly selected from our database (Table-1). All patients underwent surgery by the same surgeon, and they were followed by PSA measurement in the first 5 years each 6 months and then annually with a mean follow-up of 92.5 months. We included patients with PC and subjects that provided informed consent to participate in the study and that allowed their biological samples to be genetically tested. We excluded from the study patients who undergone adjuvant or neoadjuvant treatment. Approval for the study was given by the Institutional Board of Ethics (no. 0453/08).

**Table 1:** Demographic characteristics of 40 men submitted to radical prostatectomy to treat prostate cancer.

Age (years)		
	Mean	63
	Min - Max	41 – 79
**PSA (ng/ml)**		
	Mean	12
	Min - Max	2.0 – 37.0
	< 10 n (%)	18 (45.0)
	≥ 10 n (%)	22 (55.0)
**Stage**		
	pT2 n (%)	22 (55.0)
	pT3 n (%)	18 (45.0)
**Gleason score**		
	< 7 n (%)	14 (35.0)
	≥ 7 n (%)	26 (65.0)

All surgical specimens were formalin-fixed and totally paraffin-embedded. The slides most representative of tumor from each patient were selected by considering the area that best represented the whole tumor. Two areas from each tumor were marked with permanent ink and were included in the TMA.

The immunohistochemistry heat antigen retrieval process using citrate buffer (1mM, pH 6.0) was performed. The slides were incubated overnight at 4°C with the monoclonal antibodies specified in Table-2. The LSAB system was used for immunostaining (Dako Cytomation, CA). Color was developed by reaction with a 3, 3'diaminobenzidine substrate-chromogen solution followed by counterstaining with Harris hematoxylin. Slides were dehydrated, cover slipped and observed under a light microscope. The expression of each marker was evaluated by a single pathologist (KRML) who has considered the cases as categorically negative or positive. The results were then correlated with Gleason score that was classified as low grade (Gleason score≤6) or high grade (Gleason score≥7), pathological stage (TNM 2010) considered as organ-confined (pT2) or non-organ-confined (pT3) and pre-operative serum PSA levels <or≥10ng/mL. In addition, we analyzed the immunohistochemistry results with disease behavior considering biochemical recurrence when PSA was >0.4ng/mL.

**Table 2:** Antibodies utilized.

Antibody	Manufacturer	Diluition
MMP-9	ABnova	1:10
MMP-2	Abcam	1:100
TIMP-1	Abcam	1:100
TIMP-2	Abcam	1:100
MMP-14	Abcam	1:100
IL-8	Abcam	1:100

### Statistical Analysis

To compare the clinical characteristics of patients with PC, we used the Mann-Whitney, chi-squared and Fisher exact tests. For descriptive analysis of MMP-9, MMP-2 and its regulators expression according to pathological stage, Gleason score and PSA, we constructed a box plot, and for comparison between categories, we used the Mann-Whitney test. Statistical analysis was performed using SPSS 15.0 for Windows, and significance was set at p≤0.05.

## RESULTS

**Figure 1:**
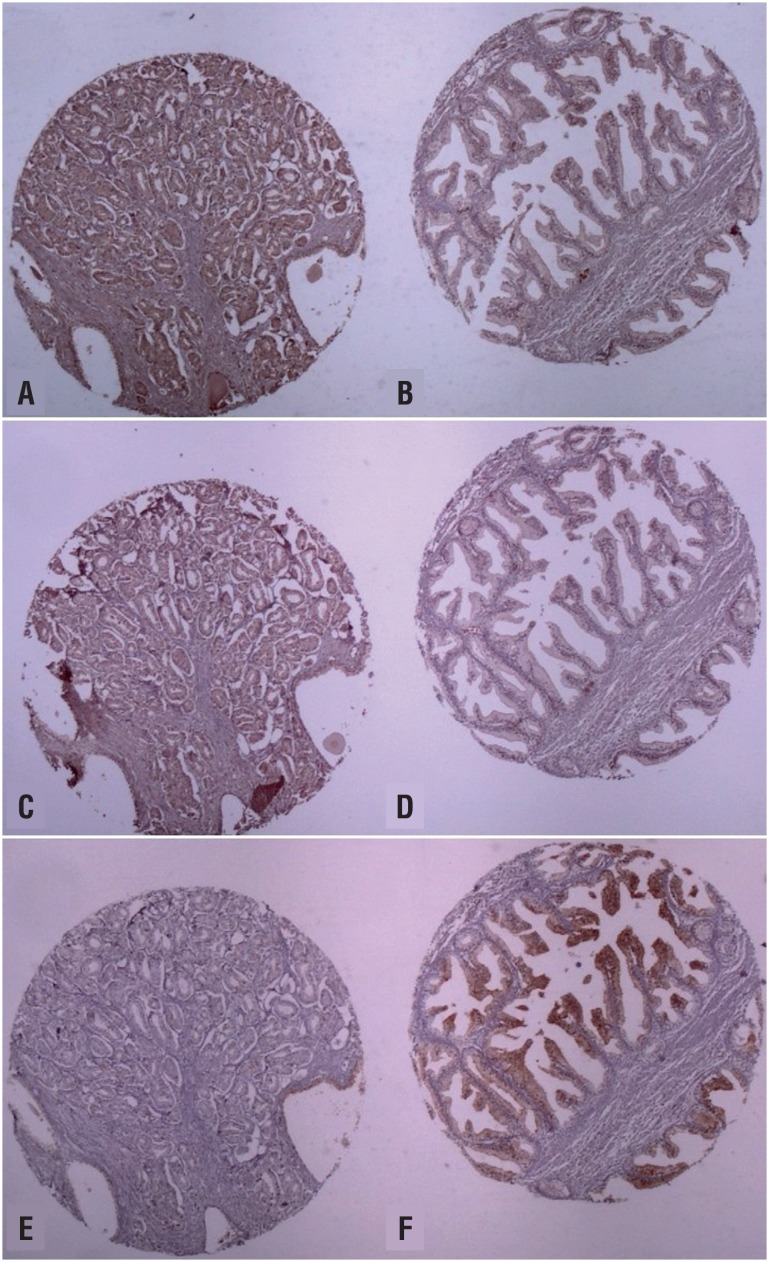
Photomicrograph illustrating immunohistochemical reactions. A: positive reaction of MMP-9 in cancer tissue; B: negative reaction of MMP-9 in normal prostate tissue; C: positive reaction of MMP-2 in prostate cancer; D: negative reaction of MMP-2 in normal prostate tissue; E: negative reaction of TIMP-1 in prostate cancer; f: positive reaction in control group.

**Figure 2:**
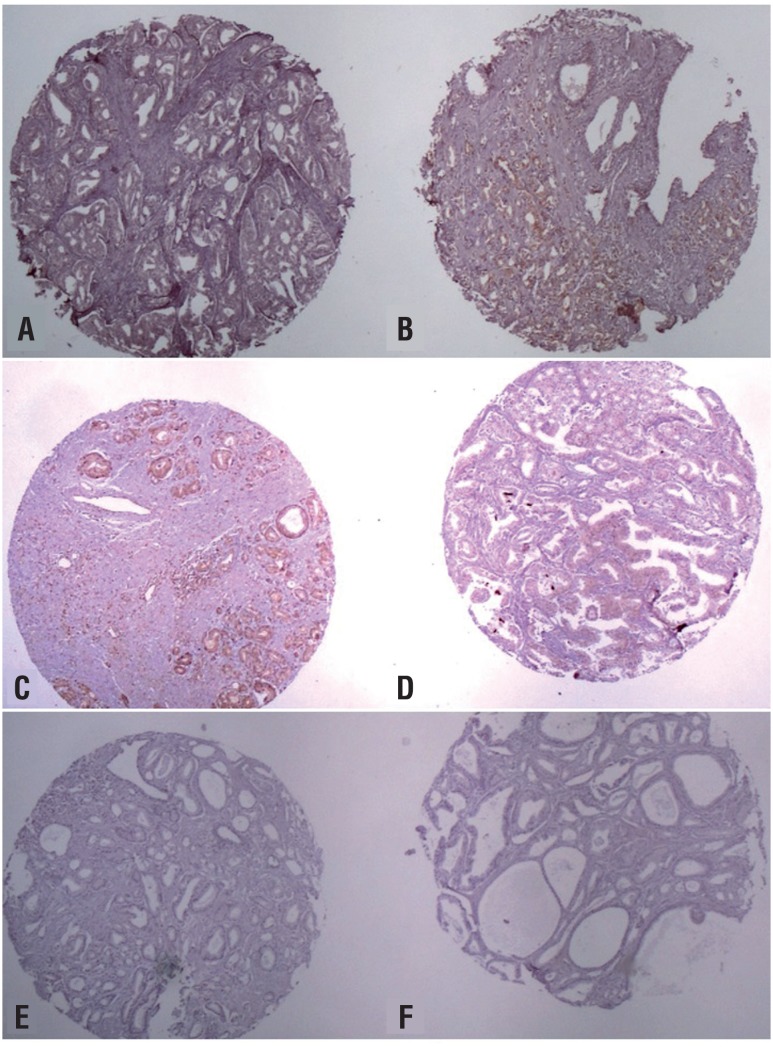
Photomicrograph illustrating immunohistochemical reactions. A: positive reaction of MMp-14 in cancer tissue; B: negative reaction of MMp-14 in normal prostate tissue; C: positive reaction of IL-8 in prostate cancer; D: negative reaction of IL-8 in normal prostate tissue; E: negative reaction of TIMp-2 in prostate cancer; f: positive reaction in control group.

The analysis of MMPs and TIMPs by immunohistochemistry was performed in a tissue micro-array conferring a standardization of the technique and microscopic analysis. Expression was categorized as positive when stain was strong or moderate and negative when there was a weak or no staining. The staining was always diffuse with no focal reaction. The expression of MMPs and their regulators were located in the cytoplasm except MMP-14 that showed interstitial positivity in some cases. MMP-9 and MMP-2 were positive in 91.4% and 77.7% of the cases respectively. TIMP-1, TIMP-2, MMP-14, IL8 were expressed by 47.2%, 0.0%, 65.7%, 63.9% of the cases respectively (Figures 1 and 2).

Analysis of the protein expression according to prognostic factors of PC is shown in Table-3. We found no statistical differences regarding the expression of any protein studied according to these prognostic variables. Statistical analysis of TIMP-2 protein was not possible, because this protein was negative in 100.0% of cases.

**Table 3:** Protein expression and Gleason score, pathological stage and PSA-value.

	Gleason Score Median (Q1-Q3)			Pathological Stage Median (Q1-Q3)			PSA-value Median (Q1-Q3)		
	< 7	≥ 7	p	pT2	pT3	p	< 10	≥ 10	p
**MMP-2**			0.777			0.288			1.000
Negative	37.5%	62.5%		75.0%	25.0%		50.0%	50.0%	
Positive	32.1%	67.9%		53.8%	46.2%		50.0%	50.0%	
**MMP-9**			0.266			0.373			0.581
Negative	66.7%	33.3%		33.3%	66.7%		33.3%	66.7%	
Positive	31.3%	68.8%		60.0%	40.0%		50.0%	50.0%	
**TIMp-1**			0.637			0.409			0.738
Negative	29.4%	70.6%		66.7%	33.3%		47.1%	52.9%	
Positive	36.8%	63.2%		52.6%	47.4%		52.6%	47.4%	
**MMP14**			0.160			0.298			0.428
Negative	15.4%	84.6%		45.5%	54.5%		38.5%	61.5%	
Positive	44.0%	56.0%		64.0%	36.0%		52.0%	48.0%	
**IL8**			0.221			0.297			0.137
Negative	23.1%	76.9%		50.0%	50.0%		30.8%	69.2%	
Positive	43.5%	56.5%		68.2%	31.8%		56.5%	43.5%	

**Figure 3:**
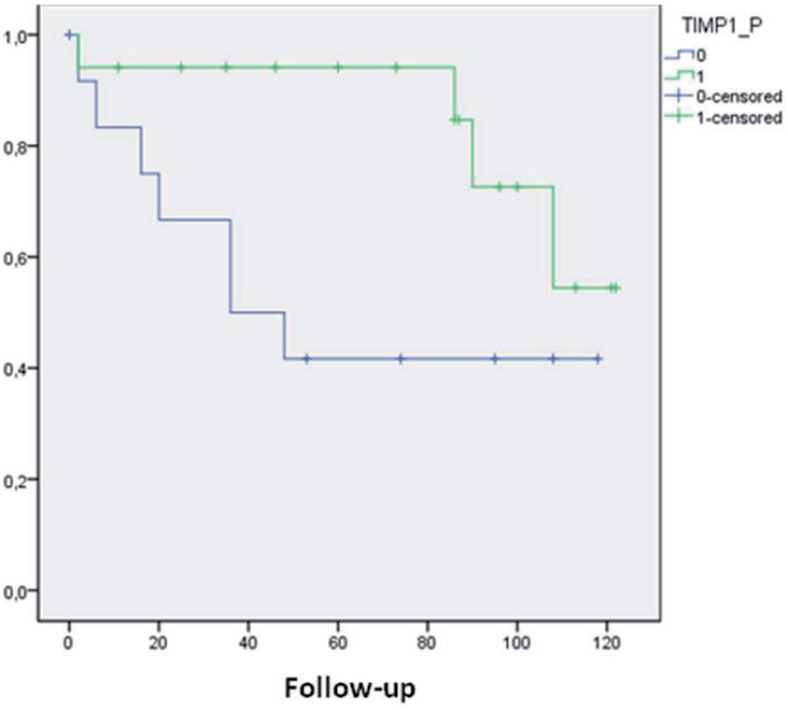
Kaplan-Meir curve of biochemical recurrence-free survival according to TIMp-1 immune-expression. Kaplan-Meier curve shows a median biochemical recurrence free survival of 105 months for patients with TIMp-1 positive (1-green line) against 62.8 months for patients with TIMp-1 negative (0-blue line).

The loss of TIMP-1 immune-expression was related to biochemical recurrence. When this protein was positive only 22.2% of cases had biochemical recurrence whereas tumor recurrence occurred in 56.3% when TIMP-1 was negative (p=0.048) (Table-4). Kaplan-Meier curve showed a median biochemical recurrence free survival of 105 months for patients with TIMP-1 positive against 62.8 months for patients with TIMP-1 negative (Figure-3).

**Table 4:** protein expression accordding to biochemical recurrence.

	Biochemical recurrence		
	No	Yes	p-value
**MMP-2**			0.248
Negative	42.9%	57.1%	
Positive	66.7%	33.3%	
**MMP-9**			0.311
Negative	33.3%	66.7%	
Positive	63.3%	36.7%	
**TIMp-1**			**0.042**
Negative	43.8%	56.3%	
Positive	77.8%	22.2%	
**MMP-14**			0.259
Negative	54.5%	45.5%	
Positive	73.9%	26.1%	
**IL-8**			0.340
Negative	54.5%	45.5%	
Positive	71.4%	28.6%	

## DISCUSSION

In the present study, we demonstrated that MMP-2, MMP-9 and MMP-14 are positive in prostate cancer and its regulators are negative in the majority of cases. Prostate cancer is the most common cancer in men, and the advanced metastatic disease is currently incurable. It is the most common male malignancy and the second leading cause of death among men in many countries, including Brazil. In the United States, 238.590 new cases and 29.720 deaths related to PC were estimated for the year 2013 (13).

Due to the lack of efficient parameters to identify potentially aggressive tumors in many cases, clinicians are frequently unable to identify patients at greater risk of disease progression. Therefore, novel molecular makers that can more precisely indicate the biological behavior and prognosis of PC are urgently needed. Extensive studies have revealed that tumor invasion, metastasis, and angiogenesis require ECM degradation, mainly by MMPs (14).

The MMP is abundantly expressed in malignant tumors, regardless of their origin and a significant correlation between the increased expression of MMP and a worse prognosis in terms of survival could be demonstrated in several cancers (15, 16). As a result, the possibility of using their expression levels as prognostic markers have been suggested.

We have previously demonstrated that MMP-9 gene is overexpressed in 82.3% of PC cases (8), and in this study we aimed to validate the gene expression results with the protein expression using immunohistochemistry. The results were confirmed since MMP-9 protein was expressed by 91.4% of cases. This phenomenon has been considered a frequent event in the process of prostate carcinogenesis, but few studies evaluated their regulators and their importance in disease progression (14).

In our cases, MMP-2 and MMP-14 were immune-expressed by the majority of PC cases (77.7%); on the contrary, the MMP-2 and MMP 14 genes were found to be under-expressed in most cases of the PC cases (5). Our results are similar to those published by Lichtinghagen et al. (2002) (17), who showed MMP-2 under-expression in prostate cancer tissue using RT-PCR. Conversely, they observed higher expression of MMP-2 at the protein level using immunohistochemistry, a result later confirmed by Brehmer et al. (18), indicating that there is a discrepancy between the levels of MMP-2 mRNA and protein expression in prostate cancer.

At the post-translational level, all MMPs are under control of specific tissue inhibitors (TIMPs) that bind proximally to the catalytic domain of MMPs, preventing substrate attachment. TIMPs are not simply regulators of MMP activity, they also have multifunctional roles that include promotion of the cell growth (9) and inhibition of angiogenesis (19). Four TIMPs have been identified. They inhibit all MMPs, forming non-covalent complexes with the active forms. Among them, TIMP-1 and TIMP-2 selectively binds pro-MMP-9 and pro-MMP-2 respectively (9, 10). Singh et al. (20) found that combined evaluation of MMP-9, TIMP-1 and TIMP-2 in plasma may facilitate clinical decision making for improved management of oral cancer. We showed that TIMP-1 and TIMP-2 are under-expressed in PC compared to BPH, and we confirmed our results, because TIMP-2 was negative in 100% of the cases and TIMP-1 was negative in 52.1%. We believe that the TIMPs control over MMPs is responsible for this discrepancy that literature has published.

Interestingly, we were able to find a relationship between the expression of TIMP1 protein and biochemical recurrence. When TIMP-1 was negative biochemical recurrence occurred in only 22.2% of the cases. Furthermore, we found a median of biochemical-free survival time of 105 months for patients with TIMP-1 positive versus 62.8 months in those where TIMP-1 was negative. At the post-translational level, all MMPs are under control of specific TIMPs that bind proximally to the catalytic domain of MMPs, preventing substrate attachment. TIMPs are not simply regulators of MMP activity, they also have multifunctional roles that include cell growth promotion (8) and inhibition of angiogenesis (19). Four TIMPs have been identified. Among them, TIMP-1 selectively binds pro-MMP-9 and is considered the main inhibitor of MMP9.

Considering all this dynamic involving MMPs and their regulators, it seems that immunohistochemistry should be more useful to study their roles in PC behavior than the mRNA profile. Also, being an easier, cheaper and widespread available method, it could be included in clinical practice as a useful prognostic parameter orienting the choice of primary or adjuvant treatment. Larger studies are necessary to confirm our statement.

## Abbreviations

BPH: = Benign prostatic hyperplasia
cDNA: = Complementary deoxyribonucleic acid
ECM: = Extracellular matrix
MMP: = Matrix metalloproteinase
BC: = Prostate cancer
qRT-PCR: = Quantitative real-time polymerase chain reaction
RECK: = Reversion-inducing cysteine-rich protein with Kazal motif
RNA: = Ribonucleic acid
TIMP-1: = Tissue inhibitor of metalloproteinases 1
TIMP-2: = Tissue inhibitor of metalloproteinases 2
IL-8: = Interleukin 8

## References

[1] Toi M, Ishigaki S, Tominaga T (1998). Metalloproteinases and tissue inhibitors of metalloproteinases. Breast Cancer Res Treat.

[2] Eissa S, Ali-Labib R, Swellam M, Bassiony M, Tash F, El-Zayat TM (2007). Noninvasive diagnosis of bladder cancer by detection of matrix metalloproteinases (MMP-2 and MMP-9) and their inhibitor (TIMP-2) in urine. Eur Urol.

[3] Liabakk NB, Talbot I, Smith RA, Wilkinson K, Balkwill F (1996). Matrix metalloprotease 2 (MMP-2) and matrix metalloprotease 9 (MMP-9) type IV collagenases in colorectal cancer. Cancer Res.

[4] Kodate M, Kasai T, Hashimoto H, Yasumoto K, Iwata Y, Manabe H (1997). Expression of matrix metalloproteinase (gelatinase) in T1 adenocarcinoma of the lung. Pathol Int.

[5] Reis ST, Antunes AA, Pontes-Junior J, Sousa-Canavez JM, Dall’Oglio MF, Piantino CB (2012). Underexpression of MMP-2 and its regulators, TIMP2, MT1-MMP and IL-8, is associated with prostate cancer. Int Braz J Urol.

[6] Sato H, Takino T, Kinoshita T, Imai K, Okada Y, Stetler Stevenson WG (1996). Cell surface binding and activation of gelatinase A induced by expression of membrane-type-1-matrix metalloproteinase (MT1-MMP). FEBS Lett.

[7] Jovanović M, Stefanoska I, Radojcić L, Vićovac L (2010). Interleukin-8 (CXCL8) stimulates trophoblast cell migration and invasion by increasing levels of matrix metalloproteinase (MMP)2 and MMP9 and integrins alpha5 and beta1. Reproduction.

[8] Reis ST, Pontes J, Antunes AA, de Sousa-Canavez JM, Dall’Oglio MF, Passerotti CC (2011). MMP-9 overexpression due to TIMP-1 and RECK underexpression is associated with prognosis in prostate cancer. Int J Biol Markers.

[9] Chen WT (1992). Membrane proteases: roles in tissue remodeling and tumour invasion. Curr Opin Cell Biol.

[10] Henriet P, Blavier L, Declerck YA (1999). Tissue inhibitors of metalloproteinases (TIMP) in invasion and proliferation. APMIS.

[11] Nawrocki B, Polette M, Marchand V, Monteau M, Gillery P, Tournier JM (1997). Expression of matrix metalloproteinases and their inhibitors in human bronchopulmonary carcinomas: quantificative and morphological analyses. Int J Cancer.

[12] Polette M, Nawrocki-Raby B, Gilles C, Clavel C, Birembaut P (2004). Tumour invasion and matrix metalloproteinases. Crit Rev Oncol Hematol.

[13] Siegel R, Naishadham D, Jemal A (2013). Cancer statistics, 2013. CA Cancer J Clin.

[14] Zhong WD, Han ZD, He HC, Bi XC, Dai QS, Zhu G (2008). CD147, MMP-1, MMP-2 and MMP-9 protein expression as significant prognostic factors in human prostate cancer. Oncology.

[15] Simi L, Andreani M, Davini F, Janni A, Pazzagli M, Serio M (2004). Simultaneous measurement of MMP9 and TIMP1 mRNA in human non small cell lung cancers by multiplex real time RT-PCR. Lung Cancer.

[16] Cho NH, Shim HS, Rha SY, Kang SH, Hong SH, Choi YD (2003). Increased expression of matrix metalloproteinase 9 correlates with poor prognostic variables in renal cell carcinoma. Eur Urol.

[17] Lichtinghagen R, Musholt PB, Lein M, Römer A, Rudolph B, Kristiansen G (2002). Different mRNA and protein expression. of matrix metalloproteinases 2 and 9 and tissue inhibitor of metalloproteinases 1 in benign and malignant prostate tissue. Eur Urol.

[18] Brehmer B, Biesterfeld S, Jakse G (2003). Expression of matrix metalloproteinases (MMP-2 and −9) and their inhibitors (TIMP-1 and −2) in prostate cancer tissue. Prostate Cancer Prostatic Dis.

[19] Cox G, Jones JL, Walker RA, Steward WP, O’Byrne KJ (2000). Angiogenesis and non-small cell lung cancer. Lung Cancer.

[20] Singh RD, Nilayangode H, Patel JB, Shah FD, Shukla SN, Shah PM (2011). Combined evaluation of matrix metalloproteinases and their inhibitors has better clinical utility in oral cancer. Int J Biol Markers.

